# Surface Topography Description after Turning Inconel 718 with a Conventional, Wiper and Special Insert Made by the SPS Technique

**DOI:** 10.3390/ma16030949

**Published:** 2023-01-19

**Authors:** Piotr Szablewski, Stanisław Legutko, Adrian Mróz, Dariusz Garbiec, Rafał Czajka, Krzysztof Smak, Bartłomiej Krawczyk

**Affiliations:** 1 Pratt & Whitney Kalisz, 4a Elektryczna Street, 62-800 Kalisz, Poland; 2 Institute of Gears Research Excellence Center, The President Stanislaw Wojciechowski Calisia University, 4 Nowy Świat Street, 62-800 Kalisz, Poland; 3 Faculty of Mechanical Engineering, Poznan University of Technology, 3 Piotrowo Street, 60-965 Poznan, Poland; 4 Łukasiewicz Research Network—Poznań Institute of Technology, 6 Ewarysta Estkowskiego St., 61-755 Poznan, Poland

**Keywords:** Spark Plasma Sintering (SPS), Inconel 718, Wiper, dry machining, surface quality

## Abstract

This paper presents a comparison of surface morphology obtained after machining Inconel 718 by the conventional insert, by Wiper insert and by using the cutting insert made by Spark Plasma Sintering (SPS). The shape of the special insert was obtained by employing Wire Electrical Discharge Machining (WEDM). The paper focuses on the description of surface topography after turning in dry and wet conditions. The performed investigation included longitudinal turning tests of Inconel 718 performed in a range of variable feeds. Surface topography measurements have been performed with the application of Nanoscan 855. The performed analysis includes a parametric evaluation of the obtained surfaces. With the Wiper insert, the *Sa* surface roughness parameter was obtained below 0.6 µm in the whole range of used feed rates. The surface roughness parameter *Sa* measured on the surface after machining by special insert depends on the cutting conditions (wet and dry machining). After, the dry machining parameter *Sa*, similar to the Wiper insert, was below 0.6 µm in the whole range of used feed rates. Unfortunately, cutting Inconel 718 using special insert with feed rate *f* = 0.25 mm/rev and cooling generated a surface with *Sa* parameter over 2 times higher than for the same feed rate without cooling, while this parameter, after turning by conventional insert, increases over 4 times using feed rate *f* = 0.25 mm/rev compared to feed rate *f* = 0.05 mm/rev during machining with cooling. This ratio is lower for conventional insert in dry machining because of sticking, which arises at the smallest feed rate according to previous research.

## 1. Introduction

Inconel 718 belongs to the group of superalloys on the nickel matrix. This material, due to its properties, such as susceptibility to hardening, low heat conductivity, high hardness and susceptibility to reaction with the tool, is difficult to machine. Due to its properties, it is widely applied in the aircraft industry [1]. Inconel 718 is a frequent object of investigation; however, although many studies on this topic have been published and knowledge about it is growing, machining it presents a significant number of problems. Tool manufacturers specializing in the production of machining nickel matrix materials assume that the lifespan of the cutting insert tool is at the level of 15–20 min. The lifespan of the cutting insert tool in machining parts of this alloy is, to a large extent, dependent on the shape of the machined parts. In the case of thin-walled elements, where vibration occurs during machining, the lifespan of the cutting insert tool is shorter. Investigations concerning the lifespan of cutting insert tools, determining the quality of the machined surface, are performed both for cutting inserts with anti-wear coatings and without them [2,3].

The cutting inserts offered by the tool producers are made by the conventional technique of metal powders sintering [4]. Spark Plasma Sintering (SPS) is an advanced sintering technique that has found increasingly wider applications [5]. In his study [6], Tokita presents the history of SPS, describing the advantages of the materials resistant to abrasion and indicating the large potential of SPS technology. One of the applications of materials made by this technique is cutting tools. Wachowicz et al. [7], in their investigation, have applied two kinds of composites for the cutting inserts for machining a three-layer chipboard.

Today, higher productivity in the manufacturing processes is required. This in turn necessitates the use of adequate cutting tools, allowing its performance to meet the quality criteria. Simultaneously, investigations are ongoing into the tool materials that will be price competitive without deterioration of the products manufactured. One of the proposals of many tool manufacturers is the application of Wiper-type cutting inserts.

Many papers show the results of investigations of the influence of cutting parameters on surface quality.

Elbah et al. [8] have noticed that increasing the feed results in the deterioration of the surface quality during the turning of a hardened steel workpiece. Similar conclusions concerning the influence of the feed rate on the surface quality have been drawn by Labuda [9] in the case of stainless steel. Karolczak et al. [10] used Wiper and conventional inserts for machining titanium alloy. They observed that the use of oil mist cooling could result in slightly low Ra and Rz parameters.

Wiper cutting insert geometry has also been discussed, with most studies showing that the feed rate has the greatest influence on the surface quality [11]. Stachurski et al. [12] have shown that when using the Wiper cutting insert, a threefold improvement can be seen in the surface quality compared to the conventional insert. Abbas et al. [13] observed that Wiper inserts improve surface quality compared to the conventional ones and help to achieve higher productivity. The advantages of Wiper inserts are more clearly visible when the cutting tool wear proceeds [14], especially when machining hard materials; in such cases, hardness has a significant impact on the tool life [15,16]. Researchers, e.g., [17,18], have shown that when lower values of the feed rate are applied, the differences between the surface quality obtained with Wiper and conventional inserts are not significant. Wiper inserts have been tested with the use of a variety of tool materials. Cutting inserts made of PCBN [19], ceramic [20] and cemented carbides [21] have been used. D’Addona et al. [22] have performed tests with Wiper inserts and obtained surface roughness similar to that obtained after grinding. Zhang et al. [23] and Abbas et al. [24] have emphasized that using Wiper insets instead of the conventional ones may result in improvements in the MRR (Material Removal Rate) with the same surface quality.

There are also researchers who have tried to improve the surface roughness with different methods. Özbek et al. [25] investigated the effects of shallow cryogenic treatment and deep cryogenic treatment after conventional heat treatment on Sverker 21 tool steel. They observed that the cryogenic treatment had positive effects on the mechanical and microstructural properties, increasing holding time for both cryogenic treatment types improved mechanical properties. However, the deep cryogenic treatment provides better mechanical properties compared to the shallow cryogenic treatment. García-Martínez et al. [26] investigated the clearance angle influence on the tool wear and tool life, cutting temperature, cutting forces and surface integrity of the dry machined part made of gamma titanium aluminide. They observed that the coating of the tool and its inclination are the most remarkable factors in the variation in surface roughness. The surface roughness of machined parts with the uncoated insert was lower than that of the coated one. Moreover, the cutting speed did not have an influence on surface roughness. In another paper, García-Martínez et al. [27] proposed a new and less polluting lubrication technique called Low Initial Lubrication condition (LIL). This technique involves applying a low quantity of oil on the workpiece and the rake face of the tool at the beginning of the machining process. Authors used this lubrication technique during turning of cupronickel 70/30 alloy. This material is widely used in marine and naval applications.

Peng and Shareef [28] have also analysed the dry turning process of a γ-TiAl alloy for a wide range of cutting conditions using a rhombic cutting insert. They found that the best results can be achieved by using a slow cutting speed. Ivchenko et al. [29] proposed a method for an effective selection of tool and cutting conditions during precise turning of C45 steel. They developed and implemented a practical tool for the quantitative assessment of the turning inserts based on the cutting process, considering the tribological interaction of the tool and machined materials, and the tool edge radius. Smak et al. [30] investigated the effects of anti-wear coatings on surface quality and dimensional accuracy during finish turning of the Inconel 718 alloy. They observed the lowest values of surface roughness parameters after machining with the cutting insert, with the coating applied by the CVD method, which is characterized by the lowest microhardness compared to the rest of the tested cutting inserts.

Based on the results of the previous investigation, the authors of the work have decided to design the shape of the cutting insert, based on the shape of insert type WNMG. The designed cutting insert has been made by the Spark Plasma Sintering technique (SPS). The insert has been subjected to comparative tests with the commercial inserts with applied anti-wear coatings.

All the tests on the influence of the Wiper type cutting inserts on the surface quality concern 2D roughness parameters. The authors have decided to compare the conventional cutting insert to the Wiper type cutting inserts and the designed special cutting insert. The aim of the research was to check whether, when machining inserts with a large value of the corner radius (Wiper and special insert) with a feed rate of *f* = 0.25 mm/rev, obtained surface roughness will be similar to that which can be obtained when turning with a conventional insert with a corner radius of 0.8 mm, using feed rate *f* = 0.05 mm/rev.

## 2. Materials and Methods

**Figure 1:**
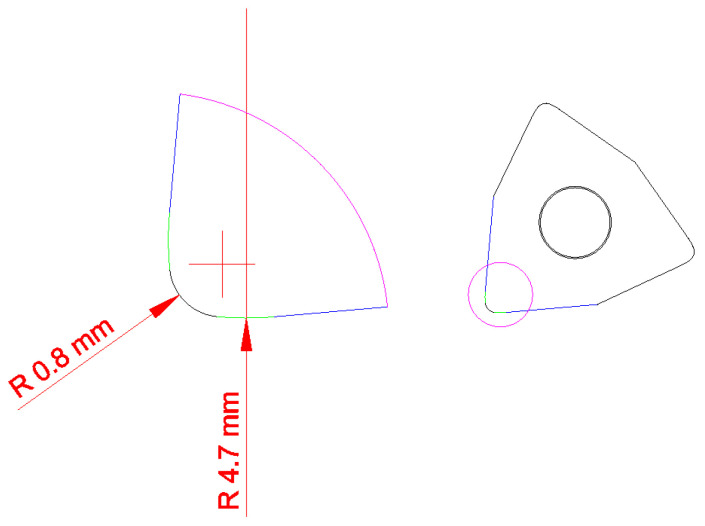
Shape of special insert prepared base on dimensions of ISO type of WNMG 080408 insert.

**Figure 2:**
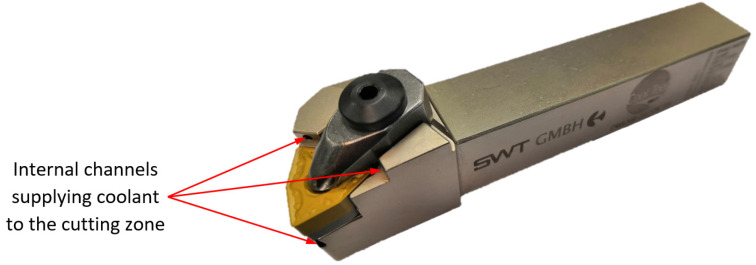
The tool holder with internal cooling channels used in the research.

**Figure 3:**
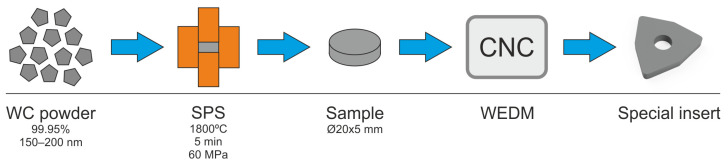
Schematic diagram of the special insert’s process preparation.

The investigation concerned the longitudinal turning of shafts made of Inconel 718. The investigations were performed on shafts with an external diameter of 130 mm and the machining length was 30 mm. WNMG 080408-FW KC5010 and WNMG 080408-UP KC5010 multi-edge inserts with a corner radius *r_ε_* = 0.8 mm and special insert with ISO type of WNMG size 080,408 SPS sintered binderless tungsten carbine were used in the tests (Figure 1). KC5010 is a type of TiAlN coating. The tool holder used was DWLNL 2525 M08, approach angle *κ_r_* = 95° (Figure 2). The tool used has internal channels to supply coolant to the cutting zone. The turning tests were performed on the CTX 2500/700 numerical lathe from DMG MORI (Bielefeld, Germany). Ecocool Global 10 lubricant was used in the tests. It is an 18% emulsion concentrate based on mineral oil and 82% of water. The tests were performed with the use of a constant cutting depth of 0.25 mm, constant cutting speed of 80 m/min and feed rates of 0.05; 0.15; 0.25 mm/rev. The cutting parameters were the same for Wiper, conventional and special inserts. Spark Plasma Sintering of WC powder with purity of 99.95% and APS of 150–200 nm (Inframat Advanced Materials, Manchester, CT, USA) was performed in an HP D 25/3 furnace (FCT Systeme, Effelder-Rauenstein, Germany). The experiments were performed in medium vacuum with a value of about 0.05 mbar. The pulsed DC with a 15 ms pulse and a 3 ms pause pattern was used for heating at the rate of 400 °C/min up to 1800 °C and then held for 5 min. The compaction pressure on the sample was kept constant at 60 MPa throughout the sintering process. The temperature was monitored by an optical pyrometer via a non-through hole in the graphite upper punch above the sample. From the spark plasma sintered compacts dimensioned Ø20 × 5 mm, cutting inserts for tests were cut by the wire electrical discharge machining (Figure 3).

Surface roughness and surface topography were measured in three locations by rotating the sample through 120°. The measurements were performed using Nanoscan 855 (Jenoptik, Jena, Germany). The Hommel Map Premium 6.2.6409 software version was used to collect and present the measurement data. Topography parameters were measured on a surface dimensioned 5.21 mm and 4.95 mm in accordance with ISO 25178 standard.

The length of the active cutting edge for the individual cutting inserts used in the investigation has been graphically determined with the use of the Catia V5R20 application.

## 3. Results and Discussion

**Figure 4:**
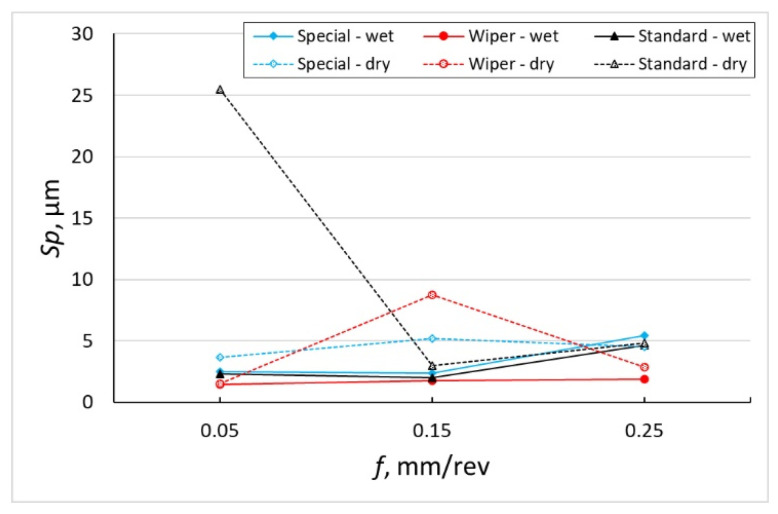
The impact of feed *f* on the variability of roughness parameter *Sp*.

**Figure 5:**
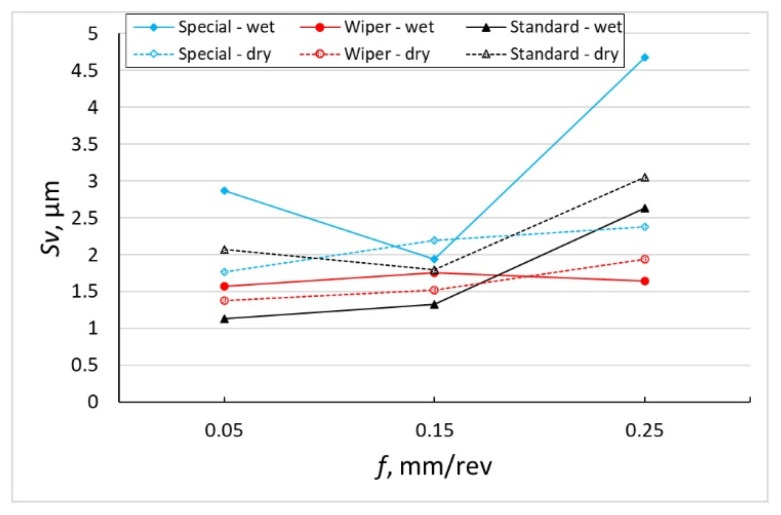
The impact of feed *f* on the variability of roughness parameter *Sv*.

The measurement results of maximum heights of the surface peaks, *Sp*, and maximum heights of the surface pits, *Sv*, both for tests performed with the use of cooling and lubrication liquid and in dry conditions are presented in the form of diagrams in Figure 4 and Figure 5. The general trend they show is that the values of those parameters grow with the increase in the feed. Although the growth of the *Sp* parameter is not so clear, it is significant for the *Sv* parameter. However, this is not a principle because, for a conventional insert after machining without coolant with the smallest tested value of the feed, *f* = 0.05 mm/rev, the average value of the *Sp* parameter amounts to 25.5 µm and the results of the individual measurements vary from 19.8 to 32.9 µm. On the other hand, the highest value of the height of the surface pit, *Sv* = 4.677 µm, has been recorded after machining with the special insert, with the application of emulsion for the highest value of the feed, *f* = 0.25 mm/rev. It has been observed that the *Sp* parameter reaches the highest values for almost all feed values under examination for the surface machined with a conventional insert without the use of emulsion, while the *Sv* parameter reaches the highest values for surfaces machined with the special insert with the application of coolant. The special insert, in machining with the use of coolant, makes it possible to obtain *Sp* parameters comparable to those obtained after machining with the Wiper insert. However, there are clear differences between the *Sv* parameters for those two cases of machining. The *Sv* parameters after machining with the special insert are about two times greater than those after machining with the Wiper cutting insert.

Krolczyk et al. [31] state that the small repeatability of measurements is a feature of the *Sp* and *Sv* parameters. The statement is confirmed in tests performed without the application of coolant. The difference between the highest and the lowest values of those parameters was as great as 13.1 µm. On the other hand, for the surfaces after machining with emulsion, the difference was up to 0.45 µm, i.e., it was almost 30 times smaller. This means that cutting Inconel 718 alloy without the application of emulsion is less stable and can generate peaks identified in [32] as sticking on the machined surface originating from the chips. The morphology of chips after turning materials used in the aerospace industry was studied by Rybicki et al. [33]. They found that the chips obtained after turning the Inconel 718 alloy with small feeds are characterized by significant losses, and these losses are a result of the so-called minimum thickness of the cut layer being too small.

**Figure 6:**
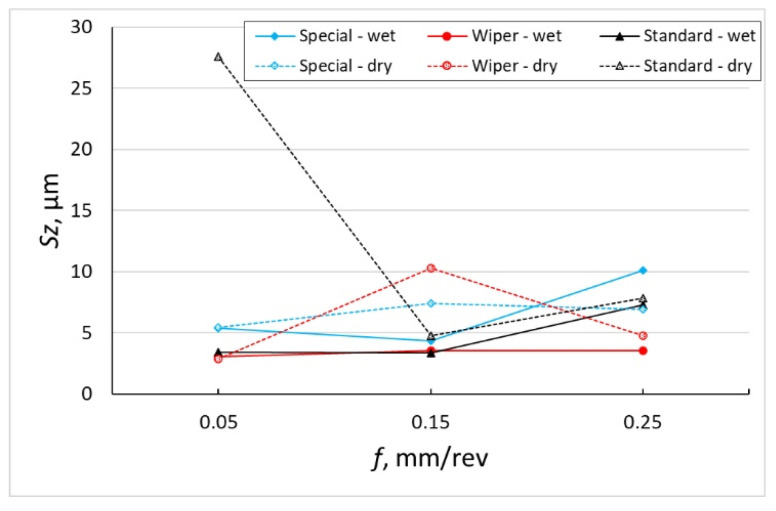
The impact of feed *f* on the variability of roughness parameter *Sz*.

The diagram of the *Sz* parameter, which is the sum of *Sp* and *Sv* parameters, shows that the smallest values have been obtained after machining with the Wiper insert with the application of coolant for all the tested values of feed (Figure 6). Similar values of that parameter have also been obtained after dry machining with the Wiper insert. An exception is a value measured on the surface after machining with the feed of *f* = 0.15 mm/rev. With that value of feed, sticking appeared on the machined surface, which has been reflected in the *Sp* parameter.

According to Królczyk [31], the cutting speed has no significant influence on the values of *Sp* and *Sv* roughness parameters. Królczyk machined the 2.4462 (DIN EN 10088-1) stainless steel and observed that the variation in these parameters due to the feed increase takes place evenly, i.e., the values of both parameters increase in an even way. Figure 4, Figure 5 and Figure 6 included in the author’s work show that machining of Inconel 718 with the Wiper insert and special insert (where contact of the cutting edge is bigger because of the large value of the corner radius compared to the conventional insert with *r_ε_* = 0.8 mm corner radius), especially without coolant, has generated *Sp* and *Sv* values which are inconsistent with this hypothesis. *Sp* and *Sv* parameters increase or decrease depending on the cutting conditions (dry and wet machining) and feed rate value.

**Figure 7:**
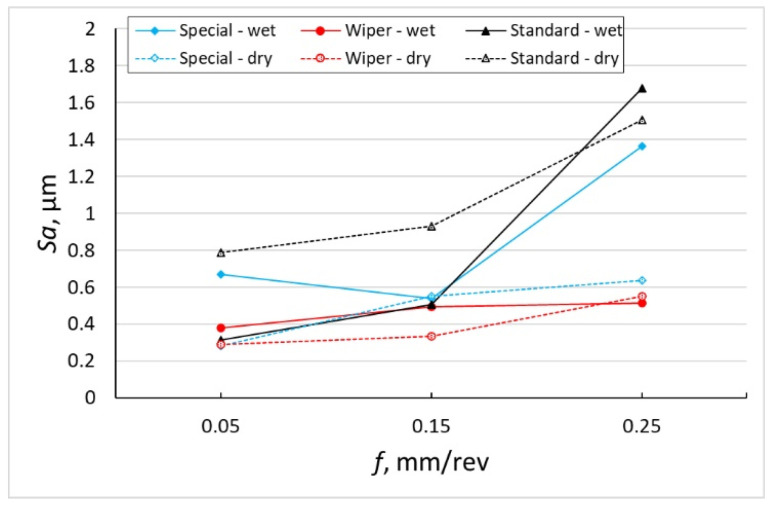
The impact of feed *f* on the variability of roughness parameter *Sa*.

**Figure 8:**
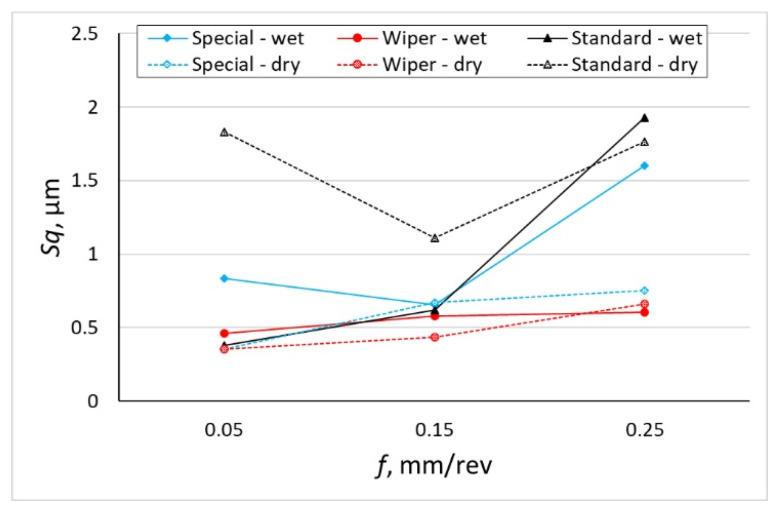
The impact of feed *f* on the variability of roughness parameter *Sq*.

The roughness parameter, *Sa* (Figure 7) and the *Sq* parameter (Figure 8) are not so much dependent on peaks and pits as the *Sp*, *Sv* and *Sz* parameters. However, for the surfaces on which sticking occurs frequently and, consequently, large numbers of peaks are recorded and thus higher values of the *Sp* parameter, larger differences between the *Sa* and *Sq* parameters have been observed. In most examined surfaces, the *Sq* parameter is about 0.15 µm larger than the parameter *Sa*. In the case of a surface machined with a conventional insert without cooling, the average value of the *Sq* parameter is 1.830 µm, while the average value of parameter *Sa* = 0.878 µm; therefore, the *Sq* value is more than twice as high.

The authors of the study expected a significant increase in the *Sa* and *Sq* parameters for the surface machined with a conventional insert with the feed of 0.25 mm/rev because it had the lowest value of the corner radius, *r_ε_* = 0.8 mm. Curious are the values of *Sa* = 1.363 µm and *Sq* = 1.600 µm obtained for the surface after machining with the special insert with the application of emulsion with the feed of *f* = 0.25 mm/rev, because the corner radius of that insert is 4.7 mm. Significant differences have been observed between the *Sa* and *Sq* parameters obtained on surfaces dry machined and machined with coolant for the lowest and the highest feed rate applied. For those feed rates, the *Sa* and *Sq* parameters for the surfaces obtained after machining with the use of coolant are twice as high as those for surfaces obtained after machining without the use of coolant.

The values of the *Sa* and *Sq* parameters obtained on surfaces machined with the special insert without cooling are comparable to the *Sa* and *Sq* parameters obtained on the surfaces after machining with the Wiper insert. Higher values of the *Sa* and *Sq* parameters of the surfaces after machining with the special insert with the coolant for the feed rates, *f* = 0.05 mm/rev and *f* = 0.25 mm/rev are related to the higher values of the *Sv* parameter. The *Sp* parameters for those surfaces are similar to those obtained on the surface machined with the Wiper insert.

**Figure 9:**
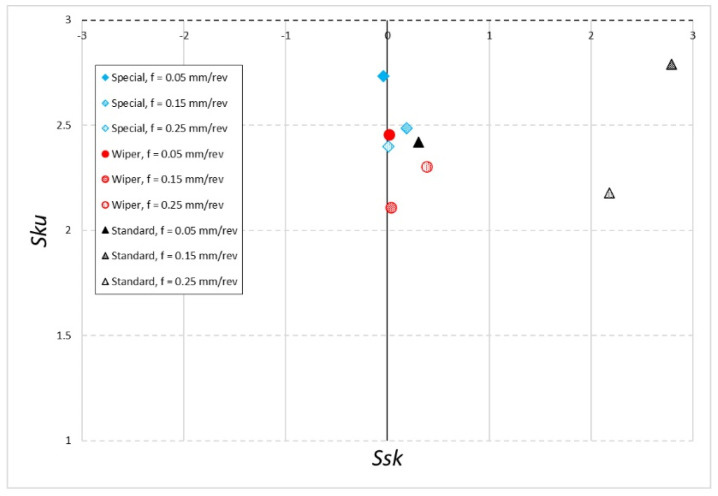
The *Sku–Ssk* map after wet machining.

**Figure 10:**
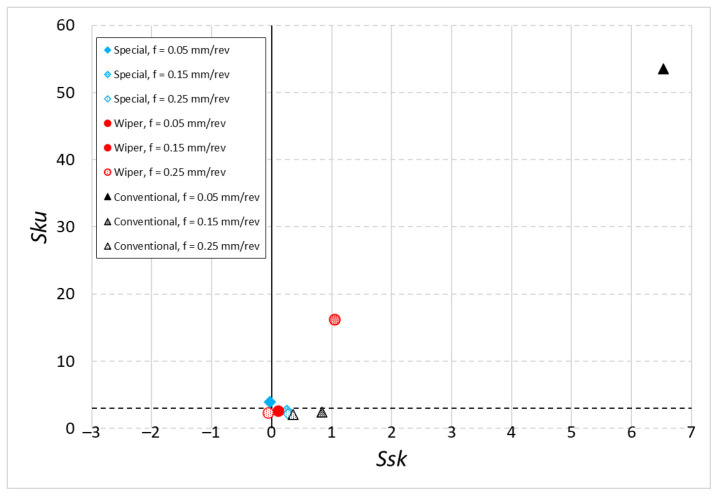
The *Sku–Ssk* map after dry machining.

Figure 9 and Figure 10 show the *Ssk-Sku* maps. The *Ssk* parameter shows the asymmetry of the surface. *Ssk* < 0 means that that pits prevail on the examined surface; *Ssk* > 0 means that vertices prevail on the surface under examination. The Sku parameter tells us whether the surface has more high vertices or deep pits for *Sku* > 3 or does not have any for *Sku* < 3 [34].

In the case of machining with coolant (Figure 9), parameter *Ssk* reaches a negative value only for the special insert with the feed of *f* = 0.05 mm/rev. This means that only under these conditions the surface has more pits than vertices and the surface shows left-side asymmetry. Other surfaces machined with the application of coolant show right-side asymmetry. The *Ssk* value close to 0 has been recorded on surfaces machined with the special insert at the feed rate of *f* = 0.25 mm/rev and with the Wiper insert at the feed rates of *f* = 0.05 mm/rev and *f* = 0.15 mm/rev. The results are significantly different from the others that have been recorded on the surface obtained after machining with a conventional insert at the feed rates of *f* = 0.15 mm/rev and *f* = 0.25 mm/rev. For those surfaces, the value of the *Ssk* parameter is above 2. This means that on those surfaces, vertices prevail. At the same time, on those surfaces, the parameter *Sku* < 3 means a regular structure of the surface. It has been observed in the case of a surface machined with a conventional insert with the use of coolant that the lowest value of parameter *Ssk* = 0.308 was obtained for the feed rate of *f* = 0.05 mm/rev. At the same time, with the same value of the feed rate for a surface machined with a conventional insert without coolant, the highest value of parameter *Ssk* = 6.530 was obtained. The *Sku* parameter of that surface also reached the highest value of *Sku* = 53.533. This provides evidence for the significant instability of the machining process.

During dry machining (Figure 10), a high value of the *Sku* parameter was observed for another examined case. A surface machined with a Wiper insert at the feed rate of *f* = 0.15 mm/rev reached the value of *Sku* = 16.170. Those surfaces are characterized by frequent sticking, which causes high values of the *Sku* parameter.

A negative value of the *Ssk* parameter for the surfaces machined without emulsion was obtained for the special insert with the feed rate of *f* = 0.05 mm/rev (similar to machining with coolant) and for the Wiper insert with the feed rate, *f* = 0.25 mm/rev.

Looking at the *Ssk-Sku* maps, one can notice that both for dry machining and machining with cooling, only for the special insert the *Ssk* values are located near the axis and parameter *Sku* < 3. However, the lowest values of parameter *Sku* were observed for the Wiper insert at the feed rates of *f* = 0.15 mm/rev and *f* = 0.25 mm/rev in the case of machining with cooling.

The study presents isometric maps for the lowest (*f* = 0.05 mm/rev) and intermediate (*f* = 0.15 mm/rev) values of the feed rate. Of course, isometric maps for all the conditions under investigation; however, for the highest applied feed rate (*f* = 0.25 mm/rev), most obtained isometric images had analogous characteristics. An exception was the isometric map obtained for the surface dry machined with the special insert. For those conditions, many high peaks were observed; the peaks are an effect of sticking appearing on the machined surface.

**Figure 11:**
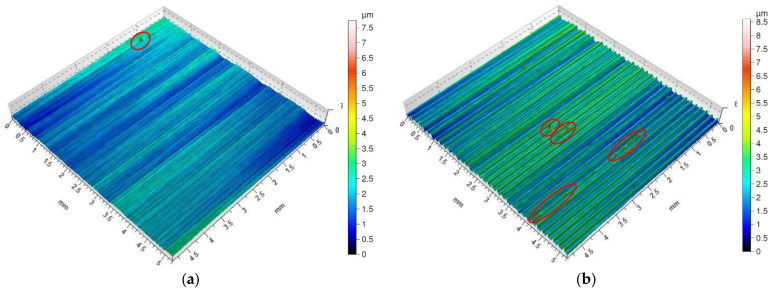

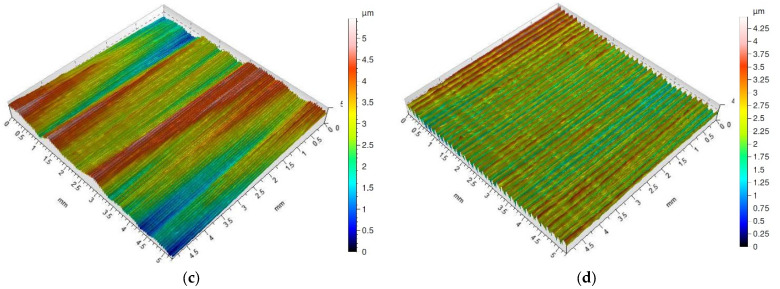
Surface topography of Inconel 718 after turning by the special insert (the sticking on machined surface is marked by red circle). (**a**) dry cutting, *f* = 0.05 mm/rev; (**b**) dry cutting, *f* = 0.15 mm/rev; (**c**) wet cutting, *f* = 0.05 mm/rev; (**d**) wet cutting, *f* = 0.15 mm/rev.

Isometric maps of the examined surfaces after dry turning and turning with cooling with the special insert are shown in Figure 11. The surfaces machined with the special insert without the use of the cooling and lubricating liquid (Figure 11a,b) are characterized by sticking, probably originating from the chip fragments. The frequency of the occurrence of sticking depends on the feed value; the larger feed the more sticking on the surface. This phenomenon has not been observed on surfaces machined with the special insert with the application of the cooling and lubricating liquid (Figure 11c,d). The surface obtained after turning with the feed of *f* = 0.05 mm/rev is corrugated and the distance between the neighboring peaks is about 1 mm. On the other hand, on the surface machined with the feed of *f* = 0.15 mm/rev, traces of the feed characteristic of the turning operation are visible.

**Figure 12:**
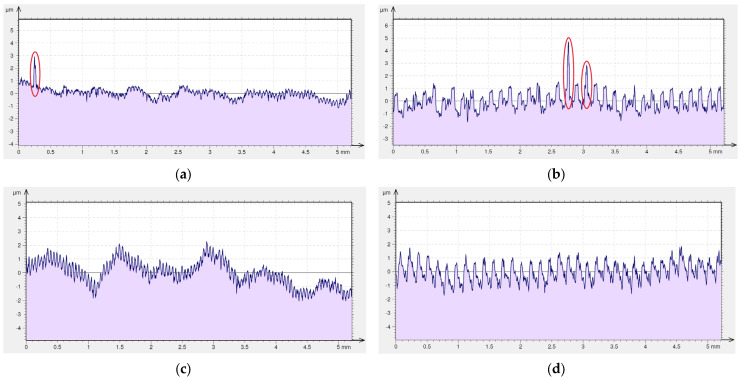
Surface roughness profile of Inconel 718 after turning by the special insert (the sticking on machined surface is marked by red circle). (**a**) dry cutting, *f* = 0.05 mm/rev; (**b**) dry cutting, *f* = 0.15 mm/rev; (**c**) wet cutting, *f* = 0.05 mm/rev; (**d**) wet cutting, *f* = 0.15 mm/rev.

Sticking, which arose on machined surfaces after dry machining with the special insert, is shown on the 2D surface roughness profiles in red (Figure 12a,b). It is higher and more often appears on surfaces machined using feed rate *f* = 0.15 mm/rev compared to feed rate *f* = 0.05 mm/rev. As the isometric views show, sticking did not appear on surfaces after machining with coolant (Figure 11c,d). This is confirmed by the 2D roughness profiles shown in Figure 12c,d.

**Figure 13:**
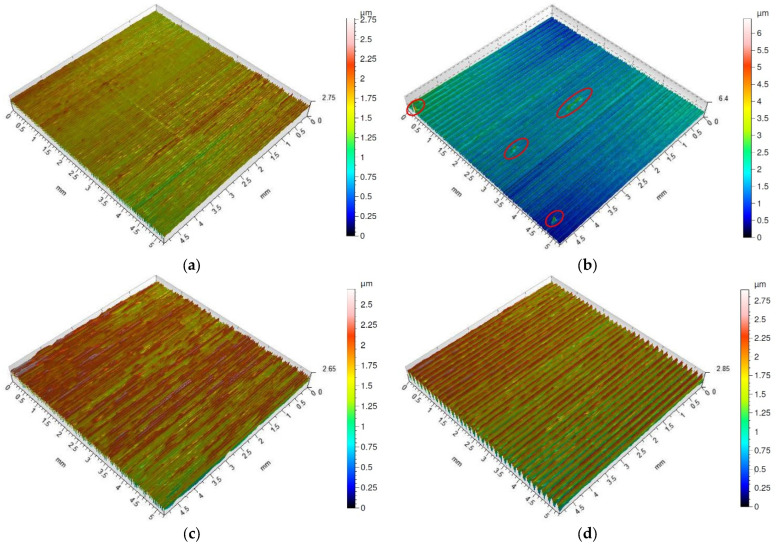
Surface topography of Inconel 718 after turning by the Wiper insert (the sticking on machined surface is marked by red circle). (**a**) dry cutting, *f* = 0.05 mm/rev; (**b**) dry cutting, *f* = 0.15 mm/rev; (**c**) wet cutting, *f* = 0.05 mm/rev; (**d**) wet cutting, *f* = 0.15 mm/rev.

The surface dry machined with a Wiper insert with the feed of *f* = 0.05 mm/rev (Figure 13a) has an uneven distribution of pits and peaks. Sticking was found on the machined surface as had been the case with the special insert. The occurrence of sticking on the surface machined with the Wiper insert has been recorded for the feed of *f* = 0.15 mm/rev (Figure 13b). Clear traces of machining characteristics of turning can be seen on that surface. A surface after machining with a Wiper insert with the use of coolant and at the feed rate of *f* = 0.05 mm/rev (Figure 13c) is characterized by irregularity, such as in the case of dry machining with the same feed value. On that surface, the prevalence of peaks over pits can be observed. The surface machined at the feed rate of *f* = 0.15 mm/rev with cooling (Figure 13d) is regular, and the distances between the neighboring peaks are similar. Curiously, the amplitude parameters obtained for surfaces obtained with the application of the cooling and lubricating liquid are comparable.

**Figure 14:**
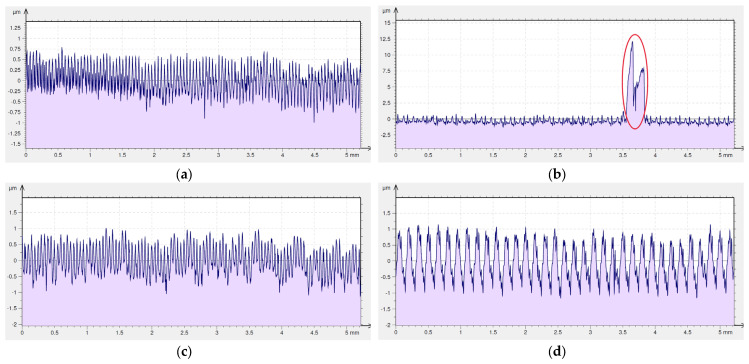
Surface roughness profile of Inconel 718 after turning by the Wiper insert (the sticking on machined surface is marked by red circle). (**a**) dry cutting, *f* = 0.05 mm/rev; (**b**) dry cutting, *f* = 0.15 mm/rev; (**c**) wet cutting, *f* = 0.05 mm/rev; (**d**) wet cutting, *f* = 0.15 mm/rev.

Similar to surfaces after machining with the special insert, 2D surface roughness profiles are presented for surfaces after machining with the Wiper insert. Unlike the special insert, sticking appeared only on the surface after dry machining with a feed rate *f* = 0.15 mm/rev (Figure 14b). The height of sticking for this surface is twice that of surfaces after dry machining with the special insert using the same value of the feed. Moreover, sticking after machining with the Wiper insert is about three times wider. Sticking did not appear on the remaining analyzed surfaces (Figure 14a,c,d).

**Figure 15:**
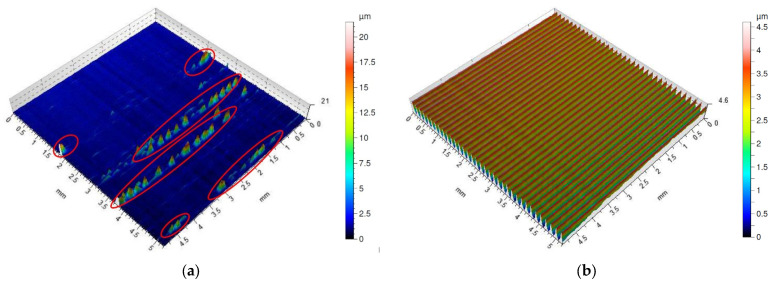

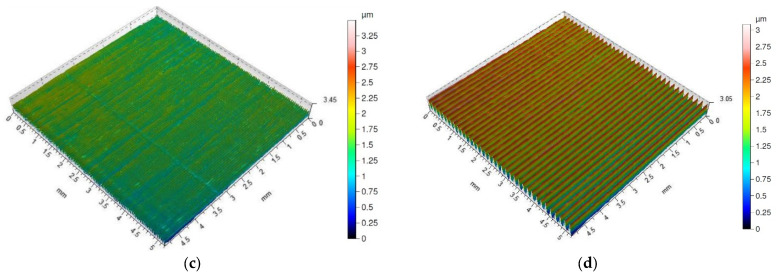
Surface topography of Inconel 718 after turning by conventional insert (the sticking on machined surface is marked by red circle). (**a**) dry cutting, *f* = 0.05 mm/rev; (**b**) dry cutting, *f* = 0.15 mm/rev; (**c**) wet cutting, *f* = 0.05 mm/rev; (**d**) wet cutting, *f* = 0.15 mm/rev.

Figure 15a shows an image of the surface made at the feed rate, *f* = 0.05 mm/rev, without cooling by means of a conventional insert. The surface is characterized by much sticking, which appears parallel to the track of the cutting insert motion. An increase in the feed rate for that cutting insert up to *f* = 0.15 mm/rev eliminates the occurrence of sticking (Figure 15b) and the distribution of peaks and pits is even. The application of cooling in turning with a conventional insert generates a surface free from sticking, both for the feed of *f* = 0.05 mm/rev (Figure 15c) and for the feed of *f* = 0.05 mm/rev (Figure 15d). Despite the higher feed value, the surface shown in Figure 15d is characterized by lower values of the amplitude parameters of the surface roughness. On that surface, one can find traces characteristic of turning with regular distances between peaks and pits. The surface machined at the speed rate of 0.05 mm/rev with the application of the cooling and lubricating liquid has the lowest value of the maximum pit depth, *Sv*, which has a value twice as high as the maximum height of the peaks, *Sp*.

**Figure 16:**
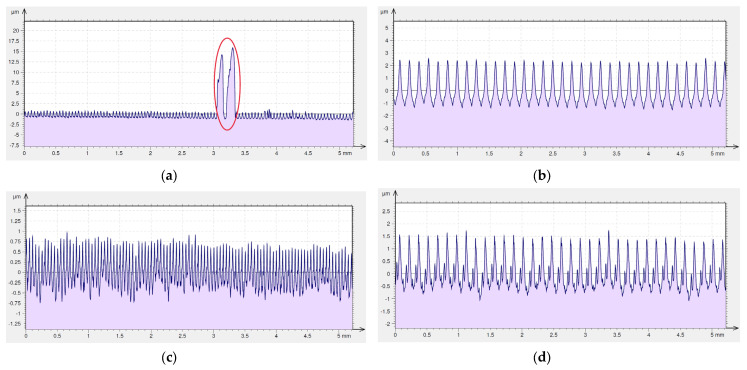
Surface roughness profile of Inconel 718 after turning by conventional insert (the sticking on machined surface is marked by red circle). (**a**) dry cutting, *f* = 0.05 mm/rev; (**b**) dry cutting, *f* = 0.15 mm/rev; (**c**) wet cutting, *f* = 0.05 mm/rev; (**d**) wet cutting, *f* = 0.15 mm/rev.

2D surface roughness profiles presented on Figure 16a–d show that machining with a conventional insert generated sticking only during dry machining with the lowest used feed value, *f* = 0.05 mm/rev (Figure 16a). A height of the sticking for this surface is even bigger than for the surface after dry machining with the Wiper insert using the feed rate *f* = 0.15 mm/rev.

**Figure 17:**
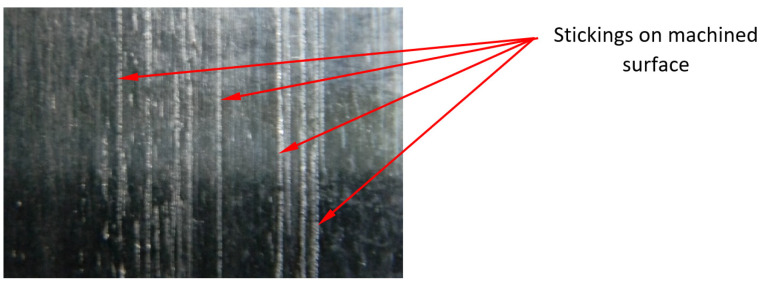
Machined surface after dry machining with conventional insert of Inconel 718, *f* = 0.05 mm/rev.

Figure 17 shows sticking which appeared on the machined surface after dry machining with the conventional insert using the smallest value of the feed rate used in previous research, *f* = 0.05 mm/rev. Similar sticking appeared during dry machining using the special insert and the Wiper insert. As shown in the isometric views and 2D roughness profiles, sticking appeared at different feeds depending on cutting inserts. The frequency and size of the sticking depended on the feeds and cutting insert type.

An analysis of the usability of the surfaces under investigation was performed based on the obtained Abbot-Firestone curves (AFC) [35]. The study presents the Abbot-Firestone curves for the selected surfaces. The selection criterion adopted was the surfaces for which *Sa* parameter was lower or equal to 0.4 µm. The authors’ decision was motivated by the fact that on the parts of aircraft engines (especially in the hot section) on the surfaces mating other elements, designers very often determine the required roughness at the level of *Ra* lower or equal to 0.4 µm

**Figure 18:**
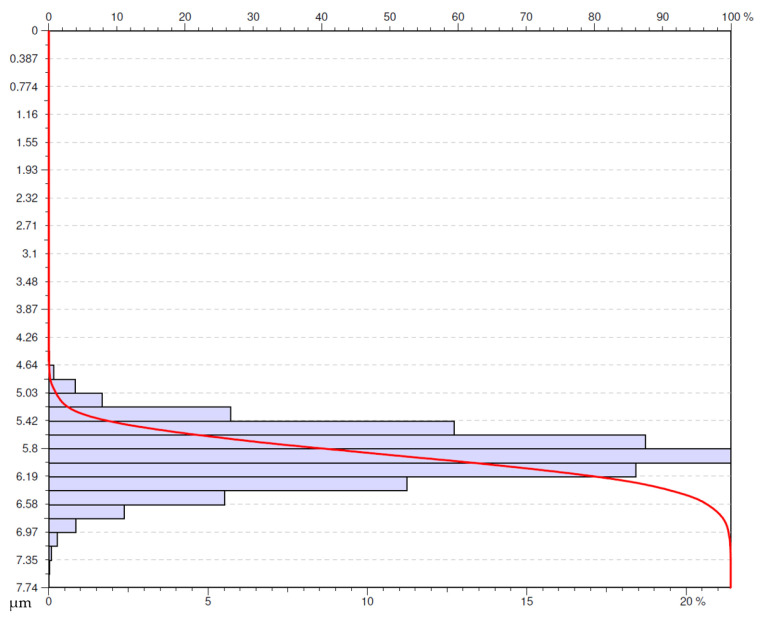
Abbott-Firestone curve after dry turning by special insert with feed *f* = 0.05 mm/rev.

**Figure 19:**
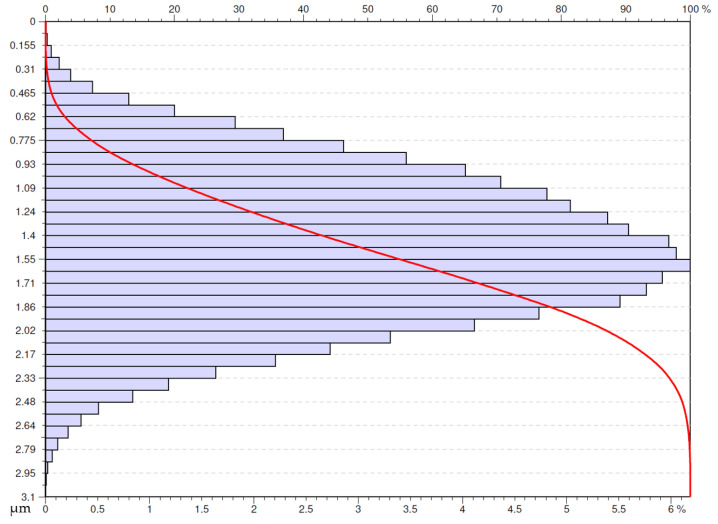
Abbott-Firestone curve after wet turning by Wiper insert with feed *f* = 0.05 mm/rev.

**Figure 20:**
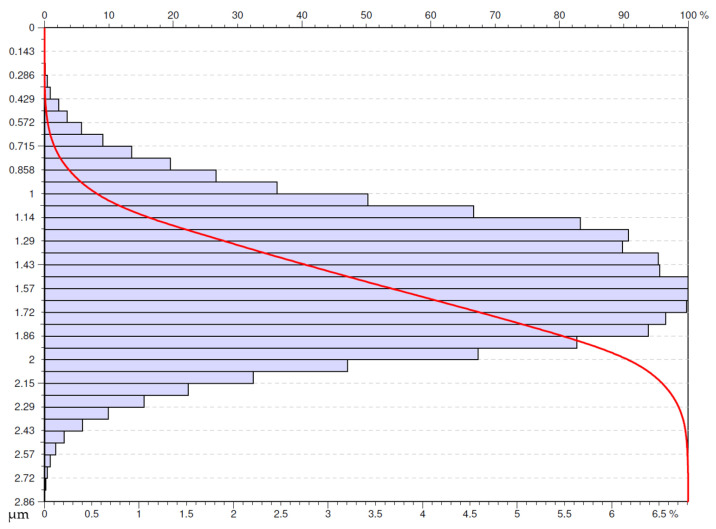
Abbott-Firestone curve after dry turning by Wiper insert with feed *f* = 0.05 mm/rev.

**Figure 21:**
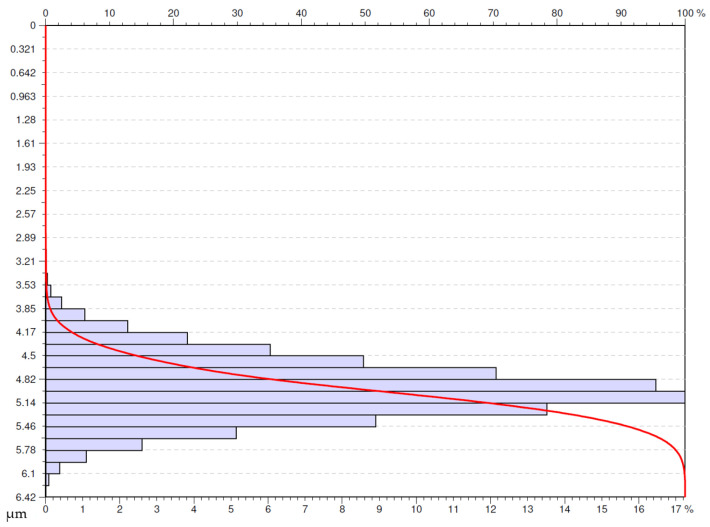
Abbott-Firestone curve after dry turning by Wiper insert with feed *f* = 0.15 mm/rev.

**Figure 22:**
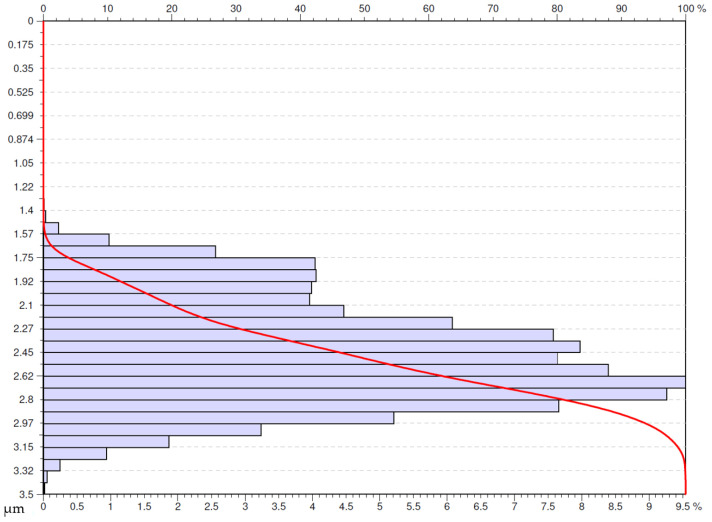
Abbott-Firestone curve after wet turning by conventional insert with feed *f* = 0.05 mm/rev.

The determined criterion was met by five surfaces under investigation (Figure 18, Figure 19, Figure 20, Figure 21 and Figure 22). One of the surfaces for which the measured value of *Sa* parameter was lower or equal to 0.4 µm was the one obtained in dry turning by a special insert at the feed rate of 0.05 mm/rev (Figure 18). The surface has even material distribution with much-concentrated distribution of the ordinate towards the higher values of the distribution. This fact provides evidence for the occurrence of high peaks on the analyzed surface.

Another surface that met the adopted criterion was the one obtained after machining with a Wiper insert with the application of the cooling and lubricating liquid at the feed rate of 0.05 mm/rev (Figure 19). This surface has a less concentrated distribution of the ordinate that the previously analyzed surface obtained with the special insert at the same feed rate. The distribution of the density of ordinates starts at low values unlike the previously analyzed surface. This means that this surface is more advantageous in respect to usability.

A similar shape of the Abbot-Firestone curve was obtained for the surface after dry turning with the Wiper insert at the feed rate, *f* = 0.05 mm/rev (Figure 20). The distribution of the density of ordinates is a little more concentrated but slightly situated towards higher values of the distribution.

An increase in the feed rate up to 0.15 mm/rev for the Wiper insert (Figure 21), under the same machining conditions, negatively influences the shape of the Abbot-Firestone curve and causes the ordinate distribution to be moved towards higher values, similar to the case of the surface obtained after machining with the special insert (Figure 18). The distribution of the density of ordinates at the feed rate of 0.15 mm/rev is more concentrated than at the feed rate of 0.05 mm/rev.

The last analyzed surface for which the condition of *Sa* lower or equal to 0.4 µm was met was obtained after turning by means of a conventional insert with the application of the cooling and lubricating liquid at the feed rate of 0.05 mm/rev (Figure 22). In that surface, the distribution of ordinates is uneven. Moreover, it is moved towards higher values of the ordinates. This means that this surface is less advantageous in respect of exploitation suitability.

If the sticking did not occur on the machined surface after turning by the special insert without the use of the cooling and lubricating liquid, causing the distribution of ordinates towards the higher values, one could state that the most advantageous shape of the Abbot-Firestone curve was obtained with the feed rate of 0.05 mm/rev. The question to be asked is: “Would the application of an anti-wear coating allow us to avoid the formation of sticking on the machined surface in the case of dry machining?” In the case of the Wiper insert, at the feed rate of *f* = 0.05 mm/rev, the application of a coating on the cutting insert has allowed us to eliminate the phenomenon of sticking formation on the machined surface. This is important information considering the limitation of the quantity of the coolant applied. An investigation of the reduction in coolant quantity in the process of turning was performed by Maruda et al. [36]. When turning stainless steel 316L, he found that the application of the MQCL with the addition of EP/AW advantageously influences the formation of the chip and its removal from the cutting zone.

## 4. Conclusions

Based on the investigation performed, the following conclusions have been formulated:

1. The SPS special insert designed by the authors of the study makes it possible to obtain roughness parameters similar to those obtained with the Wiper insert, although the special insert was not provided with an anti-wear coating.
2. The lowest values of the roughness parameter, *Sq*, were obtained when machining at the lowest applied feed, *f* = 0.05 mm/rev for all cutting inserts. The difference is that with a special insert, the lowest *Sq* value was obtained in dry machining (*Sq* = 0.355 µm and *Sq* = 0.352 µm, respectively), while for a conventional insert, the lowest *Sq* = 0.359 µm was obtained with coolant.
3. Sticking appears on the machined surface only during dry machining and the frequency of their occurrence depends on the type of inserts and the feed value. In machining with the use of the conventional insert, sticking appeared at the lowest feed used in the investigation *f* = 0.15 mm/rev. On the surface machined by the Wiper insert, sticking appeared when using the feed of 0.15 mm/rev. When machining with the special insert, the highest frequency of the occurrence of sticking was observed at the feed of *f* = 0.25 mm/rev. This means that the frequency of the occurrence of sticking is related to the active cutting edge entering the machined material.
4. The sticking caused a significant increase in the parameters, *Ssk* = 6.530 and *Sku* = 53.533. Machining with the use of a Wiper insert has generated sticking at the feed of 0.15 mm/rev, the kurtosis parameter, *Sku* = 16.170. A surface composed of many high peaks will degrade quickly when matching another surface, causing the formation of backlash (e.g., between a pin and a bush). Thus, dry cutting of Inconel 718 does not provide the desired functional properties. The roughness parameter, *Ssk*, is positive with three exceptions: after wet and dry machining at the lowest feed used for the special insert and after dry machining with the highest feed used for the Wiper insert.
5. In design drawings, roughness is often determined by the *Ra* parameter. In the aircraft industry, general roughness is often defined by the value of *Ra* = 1.6 µm. If we assume that *Ra* is equivalent to *Sa*, all the surfaces under investigation are in accordance with the requirements with the exception of one case (turning by means of a conventional insert at the feed of 0.25 mm/rev with cooling).
6. The occurrence of sticking on the machined surface after dry turning is dependent on the feed and the length of the active cutting edge. For a conventional insert with an active cutting-edge length of 0.65 mm, sticking appeared on the machined surface at the feed rate of 0.05 mm/rev. In the case of the Wiper insert with an active cutting edge length of 0.93 mm, sticking appeared on the machined surface at the feed rate of 0.15 mm/rev. In the case of the special insert with an active cutting edge length of 0.99 mm/rev, sticking appeared at all applied values of the feed; however, the largest quantity was observed at the feed rate of 0.25 mm/rev.

## Data Availability

Data sharing is not applicable to this article.
